# High brightness red emitting polymer beads for immunoassays: Comparison between trifluoroacetylacetonates of Europium

**DOI:** 10.3389/fchem.2023.1179247

**Published:** 2023-04-20

**Authors:** Daniel K. Dinga, Ewa Kasprzycka, Israel P. Assunção, Franziska Winterstein, Amina Alizade, Volkan Caliskanyürek, Dirk Blödorn, Johannes Winkle, Ulrich Kynast, Marina Lezhnina

**Affiliations:** ^1^ Institute for Optical Technologies, Münster University of Applied Sciences, Steinfurt, Germany; ^2^ R-Biopharm AG, Darmstadt, Germany; ^3^ Quantum Analysis GmbH, Münster, Germany

**Keywords:** rare earth complexes, luminescence, polymer beads, core-shell, elisa, lateral flow

## Abstract

Efficiently luminescing spherical polymer particles (beads) in the nanoscale regime of up to approximately 250 nm have become very valuable tools in bioanalytical assays. Eu^3+^- complexes imbedded in polymethacrylate and polystyrene in particular proved to be extraordinarily useful in sensitive immunochemical and multi-analyte assays, and histo- and cytochemistry. Their obvious advantages derive from both, the possibility to realize very high ratios of emitter complexes to target molecules, and the intrinsically long decay times of the Eu^3+^-complexes, which allows an almost complete discrimination against bothersome autofluorescence via time-gated measuring techniques; the narrow line emission in conjunction with large apparent Stokes shifts are additional benefits with regard to spectral separation of excitation and emission with optical filters. Last but not least, a reasonable strategy to couple the beads to the analytes is mandatory. We have thus screened a variety of complexes and ancillary ligands; the four most promising candidates evaluated and compared to each other were β-diketonates (trifluoroacetylacetonates, R-CO-CH-CO-CF_3_, R = - thienyl, -phenyl, -naphthyl and -phenanthryl); highest solubilities in polystyrene were obtained with trioctylphosphine co-ligands. All beads had overall quantum yields in excess of 80% as dried powders and lifetimes well beyond 600 µs. Core-shell particles were devised for the conjugation to model proteins (Avidine, Neutravidine). Their applicability was tested in biotinylated titer plates using time gated measurements and a Lateral Flow Assay as practical examples.

## 1 Introduction

As the rare earth molecular markers and the bead-labels discussed in the following live off the same luminescence principles, we first give a brief introduction on rare earth complexes followed by an outline on rare earth containing beads and applications. Knowledgeable readers may decide to leap over some of the introductory paragraphs.

### 1.1 Complexes

The luminescence of aromatic β−diketonates of Europium has caught the attention of scientists more than 80 years ago; a plethora of complexes and properties has been investigated in the meanwhile. The impetus of these on biomedical research is particularly impressive; dating back to the early eighties, rare earth complexes and applications have been commercialized as very sensitive immunoassays, in which the inherently long decay times of numerous complexes could be exploited to detect antibodies or hormones in minute concentrations (Siitari et al., 1983; Soini and Kojola, 1983; Bailey et al., 1984; Bertoft et al., 1984; Hemmila et al., 1984; Diamandis, 1988).

Power and success of the rare earth complexes in photonics are obviously associated with the underlying, unique luminescence mechanisms. They have been analyzed and described extensively in the literature (de Sá et al., 2000; Selvin and Lakowicz, 2003; Binnemans, 2005; Bünzli et al., 2011; Bünzli, 2016) and shall be summarized only briefly here: Excitation of, e.g., Eu^3+^ and Tb^3+^ via (intrashell) f-f transitions is possible but very inefficient due to their quantum mechanically forbidden nature. One attractive workaround is the use of rare earth complexes, in which a strongly absorbing ligand is attached to the central ion and acts as an antenna. Hence, after singlet excitation of the ligand (^1^S → ^1^S*) the ligand undergoes intersystem crossing into a triplet (^1^S* → ^3^T, promoted by spin-orbit coupling), from which intramolecular energy transfer occurs typically to (^5^D_0_ (Eu^3+^) or ^5^D_4_ (Tb^3+^), respectively, also termed resonance levels. The energetic difference (ΔE) between ^3^T and ^5^D_0_ or ^5^D_4_ is most significant: if the ligand triplet is less than roughly 2000 cm^-1^ above ^5^D_0_ (Malta et al., 1997) or ^5^D_4_ (Latva et al., 1997), energy back transfer will increasingly go at the cost of efficiency. However, if ancillary ligands with low lying triplets below the antenna are present, resonant energy back transfer may occur and can even enhance the overall quantum yield (Kitagawa et al., 2022). Additionally, non-radiative deactivation by co-coordinated, high frequency oscillators like H_2_O must be suppressed for high efficiency, which can be accomplished by the employment of ancillary ligands to avoid H_2_O-coordination. Dibenzylsulfoxide, triphenylphosphinoxide (TPPO), trioctylphosphinoxide (TOPO) or bidentate phenanthroline and bipyridine are well known examples (Malta et al., 1998; e Silva et al., 2000; Teotonio et al., 2008), whereby the bypyridine co-ligands are less efficient in the Eu-diketonates under discussion (Faustino et al., 2005; Thejo Kalyani et al., 2019). For Eu^3+^, which is in focus here, β-diketones are among the most investigated and attractive ligands, especially with fluoro-substituted alkyl groups and aromatic substituents (Binnemans, 2005; Brito et al., 2009; Wang, 2010).

One outstanding feature of Eu-β-diketonates, again a consequence of the forbidden f-f transitions, is the very long decay time of the luminescence of up to 1 ms and more, which compares to nanoseconds or less of “conventional” organic luminophores. This property is now extensively used in time-gated analyses, as the time of emission measurement can be delayed until all auto- and background fluorescence have long faded. A neat depiction of the time gated luminescence technique in biomedical labelling has been reproduced in a review by Matsumoto and coworkers, for example, which has been updated recently (Nishioka et al., 2007; Matsumoto et al., 2020), the latter including a paragraph on luminescence microscopic imaging. An added benefit of Eu-β-diketonates is the excitability of the Eu^3+^- emission in the near UV range, typically down to 350 nm, which matches the emission of modern high power LEDs and helps to avoid costly quartz optics.

Due to the stability requirements imposed by the aqueous biomatrix and the encounter with potentially adverse reactands such as enzymes, phosphates and the like, rare earth biolabelling remains a challenging task. To function reliably, the complexes must therefore possess high kinetic and chemical stabilities to withstand the biological ambience. At the same time, high brightness, i.e., high overall quantum yields and high optical absorption, must be granted, which puts another challenge to the design of the antenna-ligands. Considerable effort has thus been devoted to design stable and efficient complexes, from which numerous successful compounds have emerged as luminescent labels (Yuan et al., 1998; Yuan et al., 2001; Bünzli and Hull, 2005; Nishioka et al., 2006; Pandya et al., 2006; Nishioka et al., 2007; Bünzli, 2015).

### 1.2 Beads

The need for high complex stabilities and protection from water, especially for simple Eu-β-diketonates, had been realized at a very early stage in the search for sensitive biolabels. An elegant way to circumvent the obstacles in stability and synthesis implied above, was conquered by the imbibition of the complexes into polymer beads, as documented in a Eastman Kodak patent, already filed in 1979 (Frank and Sundberg, 1981a). Next to screening, the use of beads offers an unbeatable advantage over molecular labels in a variety of applications, which is the amplification factor: a single polystyrene bead of 100 nm in diameter and a load of 1% wt of label complexes [e.g., Eu(ttfa)_3_(TOPO)_2_, see below] would contain nominally 2000 luminescing molecules. In practice, more than one analyte molecule per bead will be needed to enable ELISA analyses for instance, of course. However, along with very high efficiencies of beads, including extinction coefficents of the complexes in excess of 50,000 M^−1^·cm^−1^, a tremendous intensity increase is possible and observed. Thus, luminescent polymer beads comprising rare earth complexes have been developed in the most interesting size range of approximately 10 to a few hundred nm; as bead matrix materials predominantly polystyrene but also PMMA, or both, are employed [(Huhtinen et al., 2008; Aikawa et al., 2016; Shin et al., 2016)]. Typically, the beads are synthesized first by micro-emulsion polymerization and then activated by incubation of the particles in rare earth solutions, although polymerization of the matrix in the presence of the complexes is also known (Ando and Kawaguchi, 2005; Desbiens et al., 2012).

The diketonate used originally was Eu(thenyltrifluoroacetylacetonate)_3_, Eu(ttfa)_3_ (Frank and Sundberg, 1981a; Frank and Sundberg, 1981b), however, soon Eu(naphthyltrifluoroacatylacetonate), Eu(ntfa)_3_, was preferred (Hemmila et al., 1984; Soukka et al., 2001; Huhtinen et al., 2005; Huhtinen et al., 2008) and commercialized (Seradyn “Fluoromax”/Thermo Fisher Scientific). TOPO, originally employed in dissociation-enhanced lanthanide fluorescence immunoassays [DELFIA^®^ (PerkinElmer, 2023)], still seems to be the ancillary ligand of choice for the incorporation into beads as well (Ando and Kawaguchi, 2005; Aikawa et al., 2016), the majority of beads being based on polystyrene, although pure PMMA is also of interest (Moudam et al., 2009; Li et al., 2013; Cardoso Dos Santos et al., 2019). Last but not least, to act as biolabels, the surface of the beads has to exhibit functional groups that can readily be conjugated to the analyte under question; numerous protocols for the conjugation have been reported, protruding carboxylates and amines being the most prominent functional groups (Petri et al., 2004; Hermanson and Hermanson, 2013; Sapsford et al., 2013; Sasaki et al., 2022): Depending on the specific method of preparation of the beads—especially the choice of the catalyst in the radical microemulsion polymerization—the beads “naturally” assume a high surface charge already (>+30 mV for 2,2′-Azobis(2-amidinopropane) dihydrochloride, AAPH, and of ca., −40 mV for Potassium peroxodisulfate, KPS). This surfcace charge is responsible for the good dispersion stability of the beads in water. The surface charge of the beads can further be altered using suitable co-polymers either in the polymerization process itself or on deposition of a shell after the concluded first polymerization of the core. We have made good experience in using acrylic acid for negative and aminoacrylate for positive charge effects. These additives are also indispensible for the subsequent coupling to proteins etc. if covalent rather than adsorptive linkage is desired.

### 1.3 Applications

After the very early recognition of the value of rare earth biolabel complexes (Soini and Hemmilä, 1979) and the proof of principle of the dissociation enhanced fluorescence immuno assay (DELFIA) (Siitari et al., 1983) for antigens, the method was rapidly extended to the analysis of proteins and antibodies, enzymes, polypeptides, DNA, hormones, drugs and to FRETs and more (Bailey et al., 1984; Parnham and Tarbit, 1987; Christopoulos and Diamandis, 1992; Selvin, 2002; Matsumoto et al., 2020). Time resolved luminescence imaging and time resolved flow cytometry (Jin et al., 2009; Chen et al., 2020) are further methods, which have of recent drawn attention. While the research on molecular labels still persists due to their unambiguous potential, bead labels have become valuable assets in quite a number of analyses in nucleic acid hybridizations and immunological and histological analyses, see the preceding paragraph.

Point of care testing (POCT) has become an important field for luminescent beads (Zhang et al., 2019), including tests for viral and bacterial infections. Although ELISA-type investigations have also been employed, Lateral Flow Immuno Assays (LFIA) are a key part of the POCT strategies, will be outlined briefly and, dealt with in the applicatory focus of this report.

LFIA is essentially a chromatographic method on a porous nitrocellulose strip (ca. 5 mm wide, 100 mm long, 0.5 mm thick) onto which at the starting part nanoparticles (gold or polymer beads) equipped with an antibody on their surface have been enriched; if the dissolved analyte (e.g., a protein) couples to the antibodies on these nanoparticles, the formed analyte-antibody-particle-composite is mobilized and flows through the strip, driven by capillary forces. After a few centimeters, the flow front has to pass a narrow line perpendicular to the flow direction, consisting of antibodies anchored to the strip. If analyte is present, the composite is captured and gives an optical response (red colour if nano-gold particles were employed, or red emission, if Eu^3+^-complexes contained in polymer beads were used). Particularly high sensitivity can be obtained, if the longevity of the excited rare states of Eu^3+^ (or Tb^3+^) is taken advantage of and time resolved luminescence from the capture line is measured. A control line, detecting particles without adhering analyte, serves as test validation. Traditionally based on gold nanoparticles (as known from pregnancy tests, for example), rare earth polystyrene and PMMA particles (beads) are increasingly emerging as notably more sensitive substitutes (Rundstrom et al., 2007; Juntunen et al., 2012; Ham et al., 2015; Song et al., 2015; Zhang et al., 2015; Shao et al., 2017; Salminen et al., 2019; Natarajan and Joseph, 2022) and are also applied to tackle the recent threat of COVID-19 (Feng et al., 2020). Prominent complexes used are aromatic diketonates like tris(2-thenoyl-3,3,3-trifluoroacetone)Eu(III)-di(tri-n-octyl phosphine oxide), Eu(ttfa)_3_(TOPO)_2_ (Aikawa et al., 2016), and tris(1-(2-Naphthoyl)-3,3,3-trifluoroacetone)Europium(III)-di(tri-n-octyl phosphine oxide), Eu(ntfa)_3_(TOPO)_2_ (Huhtinen et al., 2008; Li et al., 2013; Shin et al., 2016); in these complexes the coordination of TOPO is granting high concentrations in the polymers.

**FIGURE 1:**
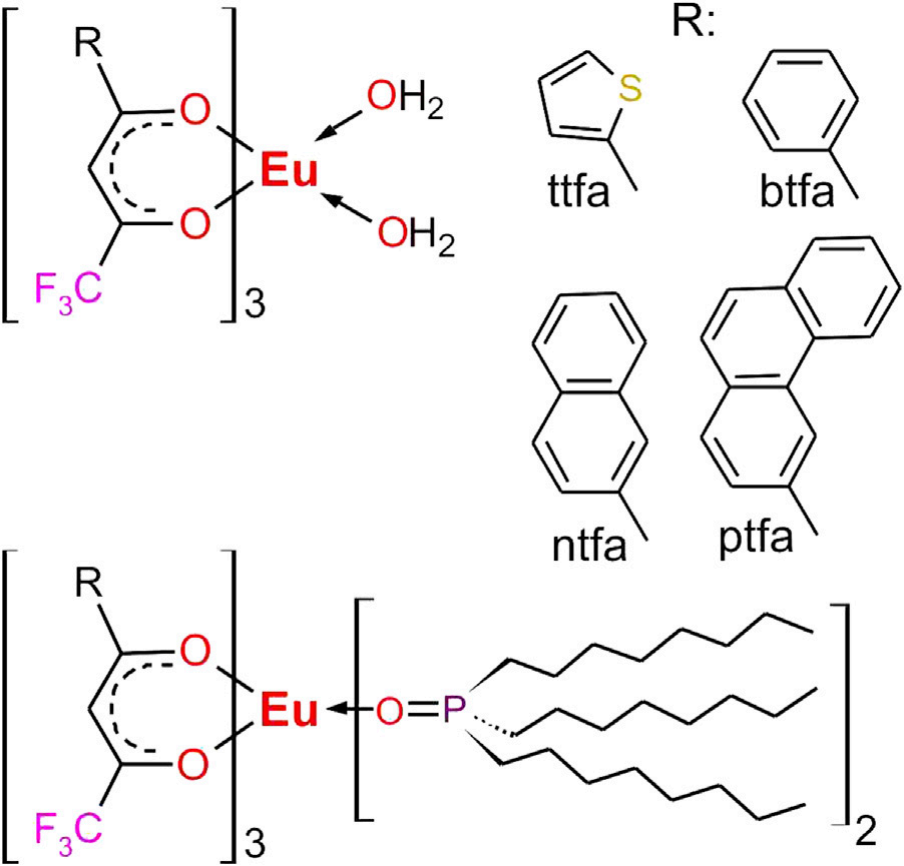
Complexes under investigation for incorporation into polystyrene beads. R = ttfa, 1-(2-thienyl)-4,4,4-trifluoro-1,3-butandion, btfa = 1-phenyl-4,4,4-trifluoro-1,3-butandion, ntfa = 2-naphthyl-4,4,4-trifluoro-1,3-butandion, ptfa = 3-phenanthryl-4,4,4-trifluoro-1,3-butandion.

Most of the aspects raised above have been documented in a large number of scientific and technical papers and patents. However, we have in recent years continued the screening for complexes suitable for incorporation in polymer beads, among them a wide variety of aromatic carboxylates and β-diketonates, with a focus on long decay times, high brightnesses and excitability in the near UV above ca. 350 nm. As may have been expected, in general aromatic carboxylates proved to be superior for green emitting Tb^3+^ beads and mentioned aromatic trifluoroforo-β-diketonates for red emitting Eu^3+^ beads. The Eu^3+^ beads form the very core of the present comparison. Some useful green emitting complexes shall be mentioned (Assunção et al., 2021; Kasprzycka et al., 2021; Rochowiak et al., 2022; Assunção et al., 2023), however, green emitting beads will be dealt with elsewhere. Next to Eu(ttfa)_3_, Eu(btfa)_3_ and Eu(ntfa)_3_, we include a novel Eu(3-phenanthtryl-trifluoro diketonate), complex, Eu(ptfa)_3_, and compare the corresponding TOPO complexes’ properties as well as polystyrene beads containing them from “one hand”; the chemical structures of above complexes are reproduced in Figure 1. A detailed procedure for evaluating Eu(ttfa)_3_(TOPO)_2_-beads in an ELISA-type as well as a lateral flow experiment is given.

## 2 Materials and methods

Spectroscopies, materials and syntheses: A detailed description of the spectroscopic apparatus employed (FTIR, Absorption, Reflectance, Excitation, Luminescence, Phosphorescence) and Fluorescence Microscopy, particle size and ς-potential determination as well as materials employed, and details for the syntheses, analyses and characterization including full IR-spectra are provided in the Supplementary Material (in the following abbreviated as “ESI”).

## 3 Results and discussion

### 3.1 Eu(diketonate)_3_(TOPO)_2_ complexes

The binary complexes Eu(diketonate)_3_(H_2_O)_2_ have all been discussed in the literature before, hence we shall refrain from commenting on them here. However, their spectral data will be given for comparison. All TOPO-complexes possess an oily consistency but show bright red luminescence under irradiation with 365 nm, whose intensity is not distinguishable with the bare eye. To cope with the oily consistencies and yet obtain comparable spectral results it was necessary to prepare powderous mixtures with KBr (1% wt of complex); these were then characterized by IR spectroscopy, reflectance, excitation and emission measurements. The IR-spectra clearly confirm the coordination of the ancillary TOPO-ligands by their C=O valence vibrations at 1,600–1,630 cm^−1^, the disappearance of the water bands above 3,000 cm^−1^, the appearance of strong alkyl C-H vibrations (2,800–3,000 cm^−1^) and the P=O valence vibration, which is redshifted from 1,146 cm^−1^ to 1,135–1,137 cm^−1^ in the TOPO-complexes. In these, the P=O frequencies coincide with C=O vibrations or appear as shoulders. Assuming c_1_ symmetry, or c_2v_ at most, all of the six C=O vibrations would be IR-active, however, due to the wealth of absorptions in that spectral regime, an unambiguous assignment of the other absorptions is almost impossible. Nevertheless, the spectra are identical to previous reports (Tran et al., 2011; Tran Thanh et al., 2014). The FTIR-spectra are reproduced in full in the ESI (Supplementary Figures S1A–D).

**FIGURE 2:**
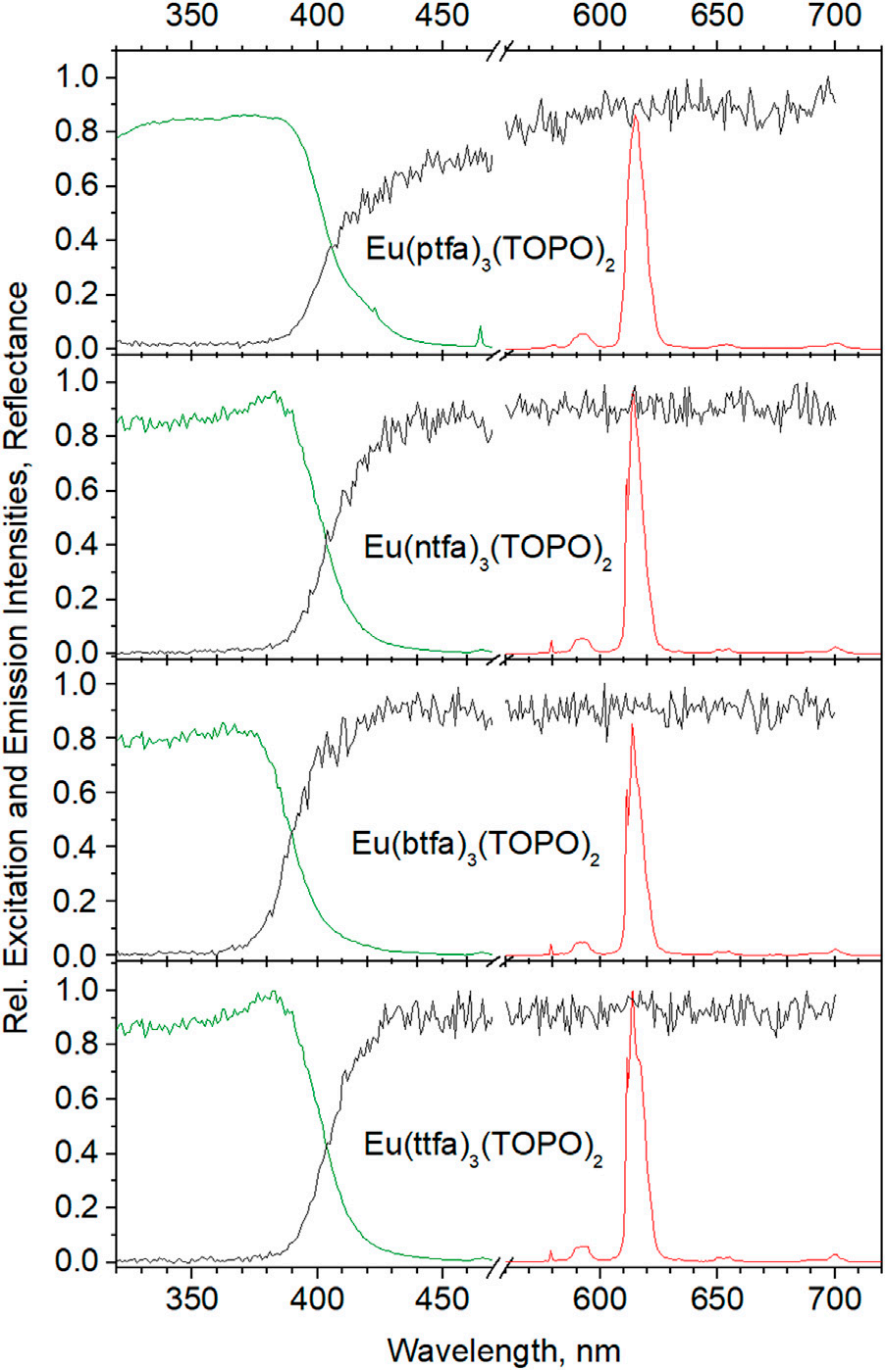
Reflectance, excitation and emission spectra of Eu(diketonate)_3_(TOPO)_2_ complexes in KBr (1% wt): F_0_→^5^D_2_ (465 nm), ^5^D_0_→^7^F_0_ (579 nm), ^5^D_0_→^7^F_1_ (593 nm), ^5^D_0_→^7^F_2_ (614 nm), ^5^D_0_→^7^F_3_ (654 nm), ^5^D_0_→^7^F_4_ (701 nm).

Reflectance, excitation and emission spectra of the TOPO-complexes as KBr-mixtures are assembled in Figure 2. The spectra are inconspicious: in emission the maxima of the hypersensitive ^5^D_0_→^7^F_2_ transitions appear at 614 nm, Eu(ttfa)_3_)(TOPO)_2_ additionally showing a Stark component at 617.5 nm as a shoulder (see caption for Figure 2 for further assignments). The complexes yield absorbances near 100% at wavelengths just below their excitation maxima. The spectral intensities are normalized to Eu(ttfa)_3_)(TOPO)_2_ with the highest excitation; it should be noted that Eu(btfa)_3_(TOPO)_2_ has its excitation maximum at 373 nm as compared to the other complexes, which optimally responded at 385 nm. The overall quantum yield of the KBr-mixtures as measured under 365 nm excitation (ligand excitation) are assembled in Table 1. Under these conditions, Eu(ttfa)_3_)(TOPO)_2_ has a slight advantage over the other complexes, but given the lower excitation wavelength of Eu(btfa)_3_(TOPO)_2_ it may be speculated that it might take the lead at optimum excitation. Other authors found yet lower overall quantum yields for Eu(btfa)_3_(TOPO)_2_ but comparably high quantum yields of Eu(ttfa)_3_(TOPO)_2_, however no data on the identity of the compounds were reported (Ohashi et al., 1990). The comparison of the decay times in KBr (fitted as monoexponentials) supports this assumption: here Eu(btfa)_3_(TOPO)_2_ has the longest lifetime of the excited state. We should also point out that mortaring the TOPO-complexes with KBr may lead to ligand exchange to some degree, and hence compromise the determination somewhat. However, the decays of the pure complexes are in fair agreement with the KBr-mixtures, except for Eu(ptfa)_3_(TOPO)_2_. We speculate that here, bromide may enter the coordination sphere, which would comply with the experimental observation that TOPO is easily lost from the complex on exposition to smaller donors. The sterically demanding phenanthryl-moiety complex seems less compatible with the equally demanding TOPO, such that it can partially be substituted, by ethanol for an example. For comparison and to further confirm that our measurements are in a good regime, we calculated the intrinsic quantum yields and radiative lifetimes, i.e., the quantum yield for intra-shell f-f–excitation and subsequent decay of the ^5^D_0_-level, which is readily accessible from corrected emission spectra using Eqs 1–3 (Werts et al., 2002):

$$\Phi_{tot}=\eta_{sens}\times \Phi_{\mathrm{int}}$$ (1)

$$\Phi_{\mathrm{int}}=\frac{\tau_{obs}}{\tau_{r}}$$ (2)

$$\frac{1}{\tau_{r}}=A_{MD,0}\times n^{3}\times \left(\frac{I_{tot}}{I_{MD}}\right)$$ (3)

**TABLE 1:** Relevant data for the complexes and corresponding beads (bead data from synthesis with 10% wt complex; final concentrations may be taken from Figure 6) *Φ*_*tot*_ = overall (experimental) quantum yields, *τ*_*obs*_ = experimental decay times; all experimental data are obtained with 365 nm excitation from powderous samples, except for the beads’ lifetimes in (aqueous) dispersions. *τ*_*r*_ = calculated radiative lifetime, *Φ*_*int*_ = intrinsic quantum yields, *η*_*sens*_ = sensitization efficiency^a^.

Complex		Eu(ttfa)_3_(TOPO)_2_	Eu(btfa)_3_(TOPO)_2_	Eu(ntfa)_3_(TOPO)_2_	Eu(ptfa)_3_(TOPO)_2_
Absorption^b^ 365 nm, %	KBr	>0.95	0.84	0.81	>0.95
	In beads	0.91	0.83	0.83	0.9
Diameter beads, nm		150	128	146	110
Quantum yield, (*Φ*_*tot*_), %	1% wt in KBr	81	78	75	72
	Powderous beads	90 (94^c^)	86	83	91
Decay times (*τ*_*obs*_), µs	Pure complex	630	675	567	449
	1% wt in KBr	649	662	594	521
	In beads	731	704	627	680
	Dispersions	717	722	633	650
Calculated^a^	*τ*_*r*_, µs	655	717	664	618
	*Φ*_*int*,_ %	99	92	89	84
	*η*_*sens*_	0.82	0.86	0.84	0.85
Zeta pot., mV	(Beads)	+47	+43	+46	+34

^a^ See text and Werts et al. (2002).

^b^ (1-Reflectance).

^c^ From synthesis with 20% wt complex.

Here, *Φ*_*tot*_ is the overall quantum yield, *η*_*sens*,_ *Φ*_*int*_ and τ_*r*_ are the sensitization efficiency, the intrinsic quantum yield and the radiative (or natural) lifetime of the emitting ^5^D_0_-state, respectively. *A*_*MD,0*_ is the spontaneous emission probability for the ^5^D_0_→^7^F_1_ transition *in vacuo* (= 14.65 s^−1^), *n* is the index of refraction of the medium (*n*_*KBr*_ = 1.5598), *I*_*tot*_ is the integrated intensity of the corrected emission spectrum, *I*_*MD*_ the (corrected) intensity of the magnetic dipole transition ^5^D_0_→^7^F_1_ and *τ*_*obs*_ the measured decay time (on ligand excitation). The calculated values are in very good agreement with the experimental data given in Table 1 and confirm the high sensitization efficiencies (>80%).

### 3.2 Beads

**FIGURE 3:**
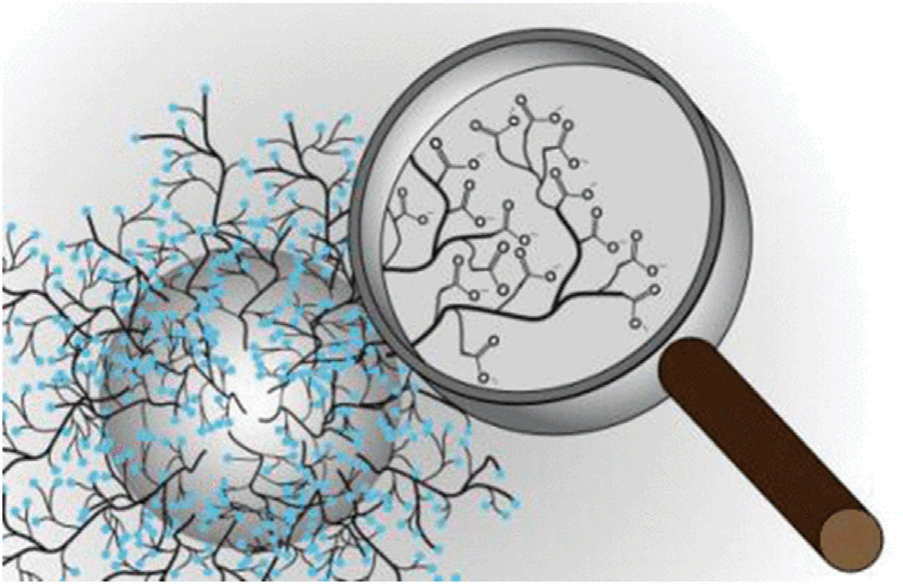
Sketch of the surface of a PMMA-bead, showing multiple and irreproducible coordination sites for ions, complexes and other species. Reprinted with permission from Andreas Hennig, Heike Borcherding, Christian Jaeger, et al., Scope and Limitations of Surface Functional Group Quantification Methods: Exploratory Study with Poly (acrylic acid)-Grafted Micro- and Nanoparticles, J. Amer. Chem. Soc., 2012. 134(19): p. 8268-8276. Copyright 2023 American Chemical Society.

In view of the applications of the beads, for this comparison we refrained from the use of additives and emulsifiers, which may interfere with subsequent coupling protocols, although size and concentrations are easier to control in the presence of additives (Ando and Kawaguchi, 2005), especially for the size regime below 100 nm (Desbiens et al., 2012). We chose polystyrene as the matrix material, as it is the dominant matrix in this context, it proved to be more compatible than polymethylmethacrylate (PMMA) with the complexes with respect to efficiencies, and not the least because in PMMA the surface control and corresponding analyses posed severe problems. We tested several analytical techniques to determine carboxlate and amine concentrations on the surface of PMMA, but the results for various methods described in the literature (Labib and Robertson, 1980; Kawaguchi et al., 1995; De Stefano et al., 2000; Dai et al., 2009; Su et al., 2010; Hennig et al., 2011) were contradictory throughout, which is most likely a consequence of the “fuzzy” surface neatly depicted in Figure 3 (Hennig et al., 2012). Levers to affect particle size and complex concentration in the micro-emulsion polymerization of polystyrene were the amounts of alcohols to some extent and the amount of complex itself. Also, for the sake of comparability for this part of the investigation, the synthesis parameters were always kept constant and the amount of complex in the synthesis set to 10% wt unless indicated otherwise. This procedure inevitably led to varying complex contents of the eventual beads as well as to varying particle sizes, but reflects the compatibility with micro-emulsion polymerization syntheses and the polystyrene matrix. The resulting bead contents are summarized in Table 1; the contents were determined by dissolution of the beads in THF and subsequent analysis by standard addition of complex and its Eu^3+^-emission signals (see ESI).

Table 1 reveals that Eu(ntfa)_3_(TOPO)_2_, efficient as it may be, is the least suitable for additive-free syntheses, and furthermore, that higher contents of complex may be possible for some species without significant alteration of their properties, Eu(ttfa)_3_(TOPO)_2_ in particular.

**FIGURE 4:**
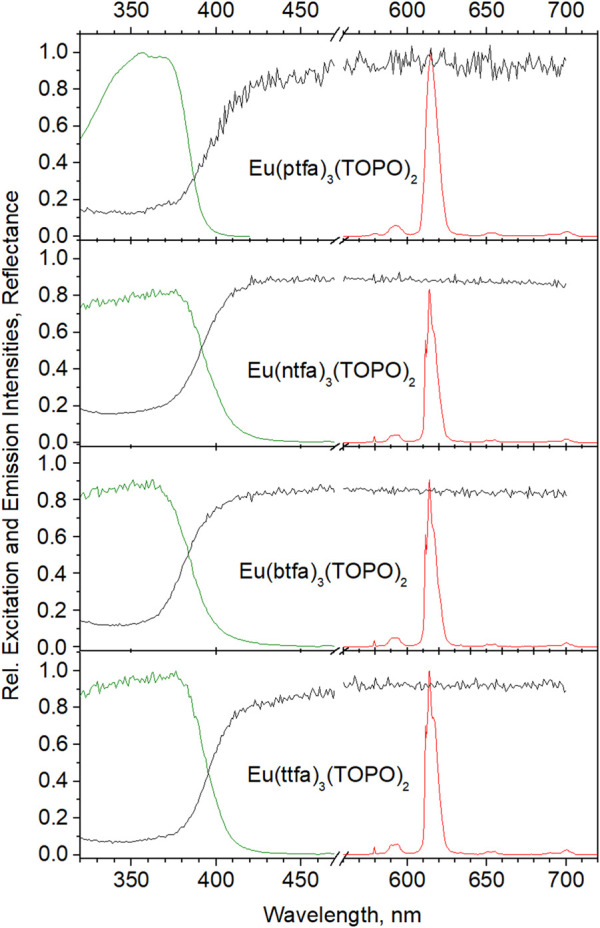
Reflectance, excitation and emission spectra of Eu(diketonate)_3_(TOPO)_2_ complexes in polystyrene beads, syntheses carried out with 10% wt relative to styrene. Spectra are normalized relative to Eu(ttfa)_3_(TOPO)_2_.

Figure 4 assembles reflectance, excitation and emission spectra of the beads from the synthesis employing 10% wt of complex. The spectral features of the complexes in KBr are practically retained completely, only the excitation maxima are shifted to lower wavelengths by ca. 5 nm. Again, Eu(ttfa)_3_(TOPO)_2_ proves to be the winner with respect to intensity at 376 nm, immediately followed by Eu(ptfa)_3_(TOPO)_2_. The somewhat lower excitation (and emission) intensity of Eu(ntfa)_3_(TOPO)_2_ is obviously a consequence of the lower concentration of the imbedded complex. All quantum efficiencies as well as the decay times are greatly increased on imbedding into the polymer (see Table 1). Brightness being a decisive factor, the beads range near the physical limit [quantum yield >90%, absorption >90%; the dimensionless term brightness is in this context (powderous samples) to be understood as the quantum yield multiplied by the absorption (1-Reflectance) as determined in an integration sphere (Wong et al., 2020)]. However, the picture changes dramatically in aqueous dispersions.

**FIGURE 5:**
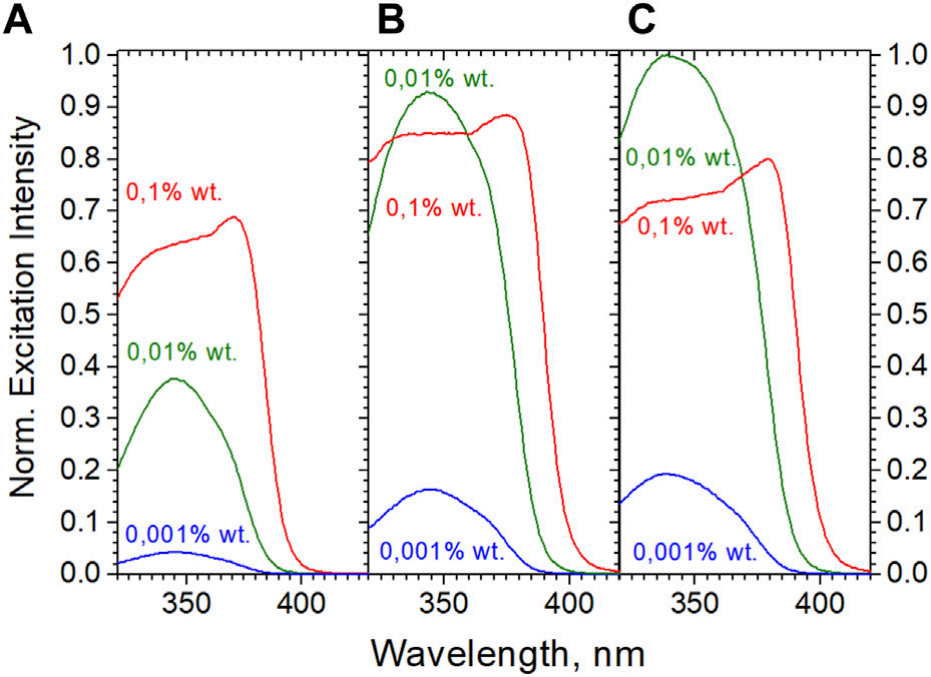
Excitation of 0.1, 0.01, and 0.001% wt aqueous bead dispersions. **(A)**: “as made” Eu(ttfa)_3_(TOPO)_2_–beads (final content of complex in beads 2.94% wt, diameter 150 nm). **(B)**: Eu(ttfa)_3_(TOPO)_2_–beads with shell from styrene-4-sulfonte (NaSS) and PMMA (“method 2”, ESI; final content of complex in beads 6.8% wt, diameter 223 nm). **(C)** Commercial Fluoro-Max—beads (201 nm).

**FIGURE 6:**
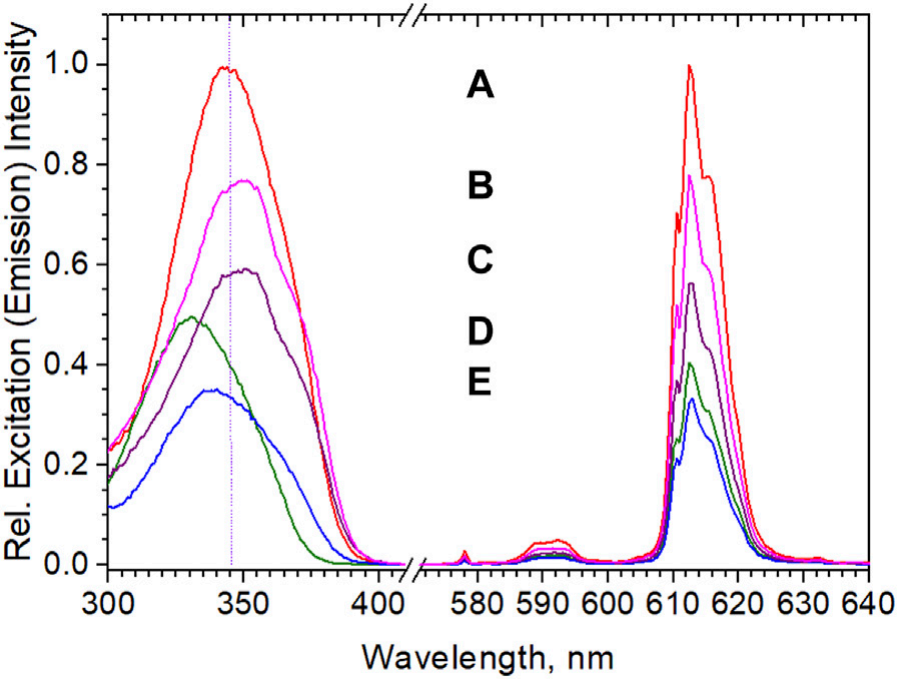
Excitation and emission spectra of Eu(diketonate)_3_(TOPO)_2_–beads in 0.001% wt aqueous dispersions, complex final content of beads in brackets: **(A)** Eu(ttfa)_3_(TOPO)_2_ (2.94% wt); **(B)** Eu(ptfa)_3_(TOPO)_2_,(1.99% wt); **(C)** Eu(ptfa)_3_(TOPO)_2_ (1.2% wt); **(D)** Eu(btfa)_3_(TOPO)_2_ (2.67% wt); **(E)** Eu(ntfa)_3_(TOPO)_2_ (0.82% wt). Spectra are normalized relative to Eu(ttfa)_3_(TOPO)_2_.

Figure 5 shows the Eu(ttfa)_3_(TOPO)_2_-beads in 0.1, 0.01, and 0.001% wt aqueous dispersions. The excitation maxima shift from 374 nm in powderous beads to 345 nm on diluting the 0.1% dispersion to 0.01%, further dilution does not alter the excitation maximum. At the same time, the intensity drops by a factor of 1.3 only rather than a factor of 10, while the second dilution step (0.01–0.001) affords an intensity decrease by a factor of 8. These factors are clearly due to inner filter effects at too strong absorbances and scattering by the beads. This behaviour is hard to unravel quantitatively in turbid media: next to the inner filter effect the spatial directions of scattering of both, excitation and emission are wavelength-dependent (Rayleigh-Gans-Debye scattering). Thus, we were not able to determine the efficiencies in dispersions reproducibly and refrain from reporting these. However, the decay times (Table 1) do not change with the concentration of the dispersions and are in good agreement with the data reported for the powderous beads. This holds true for a commercial dispersion as well (right), where the discontinuity of the intensity in the dilution series is even more pronounced [core-shell-beads (5b) and Fluoro-Max-beads (5c) are elucidated in detail in the following paragraph]. It therefore seems safe to assume that the quantum efficiencies established for the dry beads prevail in dilute dispersions. Figure 6 shows the comparison of the various bead-imbedded complexes in 0.001% wt dispersions, Eu(ttfa)_3_(TOPO)_2_ here too exhibiting the highest efficiency. It is worth pointing out that the high positive ζ-potentials of the beads (Table 1) synthesized by the protocol given in the ESI are responsible for the stability of the dispersions.

### 3.3 Surfaces

For eventual applicability, next to the stability of the dispersions, the surface of the beads needs to carry functional groups suitable for coupling to the analyte. We pursued several strategies to accomplish either amino- or carboxylate-surfaces employing co-polymers in one-pot-syntheses or as surface layers. For this purpose, p-aminostyrene, vinyl benzyl amino hydrochloride, vinylbenzylchoride and subsequent aminolysis, acrylic acid, divinylbenzene, 2-aminoethylmethylacrylate (AEMH) and others were used. All attempts of exploiting the ease of one-pot-syntheses failed insofar as they were notoriously accompanied by losses in efficiency. Although for our purposes, negatively charged carboxylate-surfaces proved to be more successful, in principle, positively charged amine-surfaces can be obtained as well, of course; experimental details for a succesive core-shell procedure for amino-beads are provided in the ESI for completeness.

**FIGURE 7:**
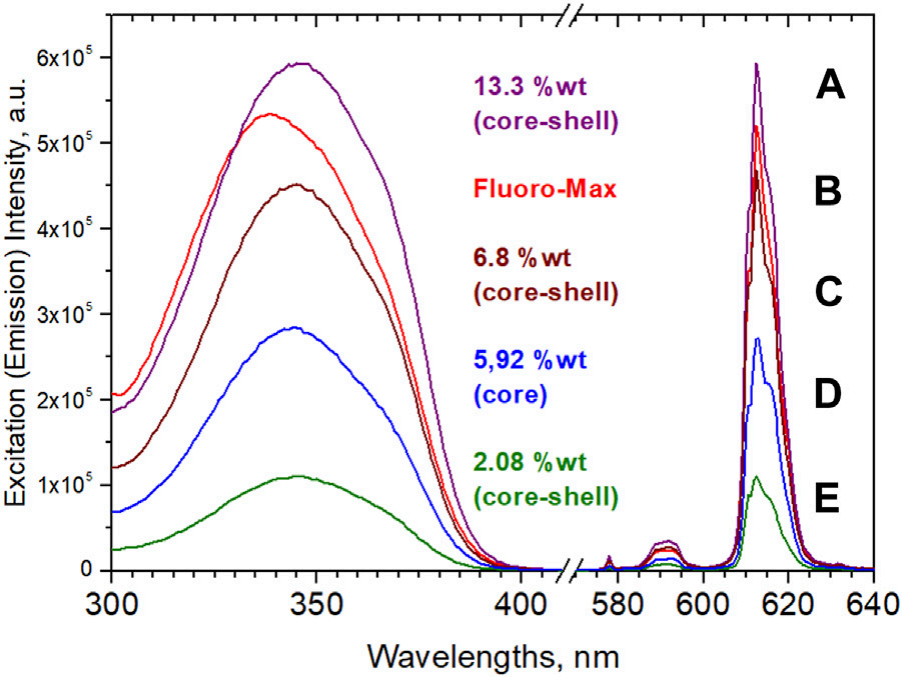
Excitation and emission of aqueous 0.0025% wt dispersions. Note that **(D)**, “naked”, and **(C)**, core-shell, have practically the same complex contents. Particle sizes were 190 nm **(E)**, 150 nm **(D)**, 223 nm **(C)**, 201 nm **(B)**; agglomerated-bimodal 334 nm and 147 nm **(A)**. **(B)** is the producer’s value for Fluoro-Max.

**FIGURE 8:**
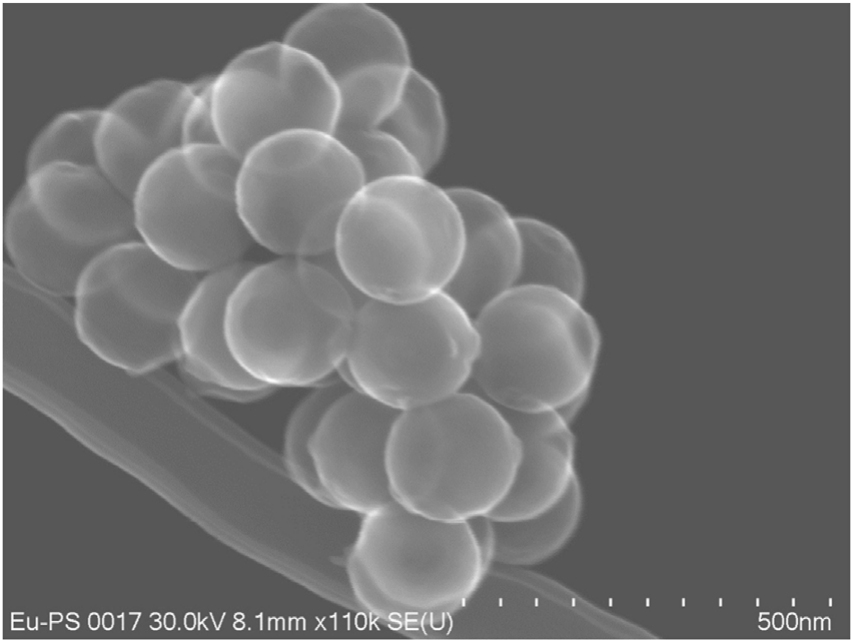
Core-shell beads with imbedded Eu(ttfa)_3_(TOPO)_2_ with a carboxylate shell (core shell method 1); secondary emission image from a Hitachi 8230 Field Emission Gun Scanning Electron Microscope, 30 kV).

For the investigations to follow, Eu(ttfa)_3_(TOPO)_2_ was chosen for its obvious suitability with respect to physical and optical properties as evident from Table 1. Two principle methods for the preparation of negatively charged core-shell beads were developed and tested (ESI, method 1 and method 2), both methods utilizing potassium peroxodisulfate (KPS) for the core, which is very compatible with the Eu-complexes. In method 1, after the formation of the core, which takes approximately 1 h for the desired sizes, a 1:1 mixture of acrylic acid and methylmethacrylate was added without previous isolation of the core. The subsequent second polymerization step was initiated with ACVA-initiator (4,4′-azobis-(4-cyan-veleric acid) and continued for 4 h. A slight drop in quantum yield (see Table 2; Figure 7) indicates that the ACVA-initiator or acrylic acid may be able to diffuse into the core, where it destroys part of the complex, an observation that we had seen in numerous other experiments before, e.g., in core-syntheses using ACVA as the initiator. The quality of core shell beads from this method may also be inspected in Figure 8.

**TABLE 2:** Properties of carboxylated core-shell beads.

Eu(ttfa)_3_(TOPO)_2_ core-shell beads	Method 1	Method 2
Complex content theor., (weight-in) experimental	10	20
	2.08	6.80
Diameter beads, nm	190	223
1-Reflectance (absorption), 365 nm, %	89	95
Qantum yield, 365 nm, %	93	90
Decays, µs, dry beads	700	768
Decays, µs, 0.01% dispersion	697	663
Zeta potential, mV	−46.3	−50.8

The somewhat lower quantum yield and absorption from method 1 prompted us to develop method 2, which was able to carry 20% wt of the complex in the synthesis and led to beads containing 6.08% wt. For method 2 we employed a mixture of acrylic acid, styrene-4-sulfonic acid sodium salt (NaSS), and methylmethacrylate for the shell, strictly avoiding acidic pH-values by neutralization and reaction in MOPS-buffer. The main properties of the beads obtained by the two methods are collected in Table 2. Hence, the best emerging shell materials were acrylic acid/methylmethacrylate and acrylic acid/styrene-4-sulfonate/methylmethacrylate, as described below. We should mention that very bright core-shell beads with yet higher complex-content (13.3% wt) could be obtained (see Figure 7). However, these were partly agglomerated as shown by a bimodal size distribution (see the description for method 1 and Supplementary Figure S2 in the ESI for more details).

It is at this stage useful to compare the bead’s properties with commercially available particles. Among the commercial beads, the reference gold standard for our purposes was Fluoro-Max with Eu^3+^-chelate imbedded particles of 200 nm diameter and roughly 500,000 surface carboxylate groups per bead: the so called “parking area” (area available for one carboxy group) varies from batch to batch between 22 and 32 Å^2^ (ThermoFisher-Scientific, 2023). Determinations of active surface groups of our own beads typically ranged around 450,000 –COOH per bead (parking area 25 Å^2^). The procedure for analysis involved EDC/NHS activation, followed by reaction with hexylamine and titration of hexylamine remaining in the mother liquor with o-phthalaldehyde (Roth, 1971) (details in the ESI). In any case, these numbers of surface carboxylates by far exceed the number of, e.g., proteins that can be attached for sterical reasons: assuming a diameter of 5 nm of a folded protein, a maximum of roughly 1,600 protein molecules can be accomodated. Due to the high price (ca. 10 €/mg; furthermore, presuming a concentration of 2% wt of complex in the beads, only 20 µg would be contained) it was prohibitive for us to completely analyze the dispersions in all respects. Therefore, the exact amount of complex and its chemical nature as well as the radial composition of the beads is not known to us with certainty, but all observations indicate that roughly 2% of complex (based on the calibration of the emission intensity after dissolution in THF) are contained. The stability of the beads in DMSO further indicates that the polystyrene is probably a crosslinked polymer, as opposed to the beads prepared for this work, which showed considerable swelling or dissolution. Furthermore, while the emission spectra of the Fluoro-Max-dispersions coincide with practically all the diketonates and provide no positive proof, the decay time of 653 µs and the excitation maximum at 338 nm suggest that the complex might be Eu(nta)_3_(TOPO)_2_ (see Figure 6; Table 1). Figure 7 shows a direct comparison of Eu(ttfa)_3_(TOPO)_2_-beads without shell, core-shell beads after “methods 1 and 2” and Fluoro-Max in 0.0025% dispersions, where inner filter effects are absent.

Figure 7 confirms that the brightness seems to scale with the concentration of complex in the beads. But it also reveals that the equipment with a shell proved to not only be crucial for subsequent coupling reactions, but also for the optical performance of the beads in dispersion, which was another important lesson to be learned: while all dry bead powders show high efficiencies, in aqueous dispersions corresponding core-shell-beads exhibit a considerable intensity-increase over “naked” particles (see Figure 7D, “naked,” and Figure 7C, core shell). Screening of surface-complexes or surface-near complexes against water, prevents quenching and decomposition. Hence, the brightness of “naked” beads amounted to only 60% of the core-shell beads. The quenching of surface-complexes may even affect parts of the core efficiency, if energy transfer from core-excited species to outer complexes occurs. The fact that the shelled beads (C) in Figure 7 with almost ideal quantum yield, near complete absorption and an optimized shell are yet still outperformed by Fluoro-Max beads is astounding on first sight. Either the complex content in Fluoro-Max-beads is notably higher (which contradicts our analyses), a different complex is occluded (which contradicts spectra and decay times) or the shell is more perfect (e.g., crosslinked polymer as the shell). Additionally, reduced scatter, i.e., stronger absorption, due to a higher concentration of complexes in surface-near areas may also contribute, although post-infiltration experiments to simulate this were not successful with our beads.

Finally, we should mention that the commercial dispersion may contain larger particle due to agglomeration, as suggested by fluorescence microscopy (see Figure 10, “Fluoro-Max”), possibly originating from prolonged storage or insufficient cooling.

### 3.4 Protein conjugation, ELISA and LFIA

The evaluated optical and surface properties obviously require further characterization to decide on the qualification of the beads for immunological analyses. We have therefore exemplarily coupled Avidine and Neutravidine as model proteins to the (carboxylated) beads, with which the very strong bonding to Biotin can be exploited. Neutravidine was chosen for the lower isoelectric point (i.e.p. = 6.4), as Avidin (i.e.p. = 9.4) might give rise to stronger adsorptive interaction rather than covalent coupling to the negatively charged beads and falsify the general picture for proteins with lower, i.e.p. The coupling routine proceeded via EDC-NHS activation as laid out in detail in the ESI and subsequent coupling with the proteins. A parallel experiment was run with non-activated beads onto which the proteins can only be adsorbed rather than covalently linked, thus informing on non-specific interaction, which would compromise the analysis. The number of Neutravidin molecules coupled to the surface was determined by titration with 4′-hydroxyazobenzene-2-carboxylic acid (HABA) as described in the ESI. HABA forms a weak complex with Neutravidin; on addition of Biotin it is released and can be determined photometrically in solution. This analysis showed that roughly 2300 Neutravidin molecules resided on the beads corresponding to 88% coupling efficiency. This number is fairly close to the maximum amount of ca., 2,700 molecules/bead as estimated from the hydrodynamic diameter of 7.4 nm for Neutravidin (Langer et al., 2014), even though we had refrained from employing excess Neutravidin.

**FIGURE 9:**
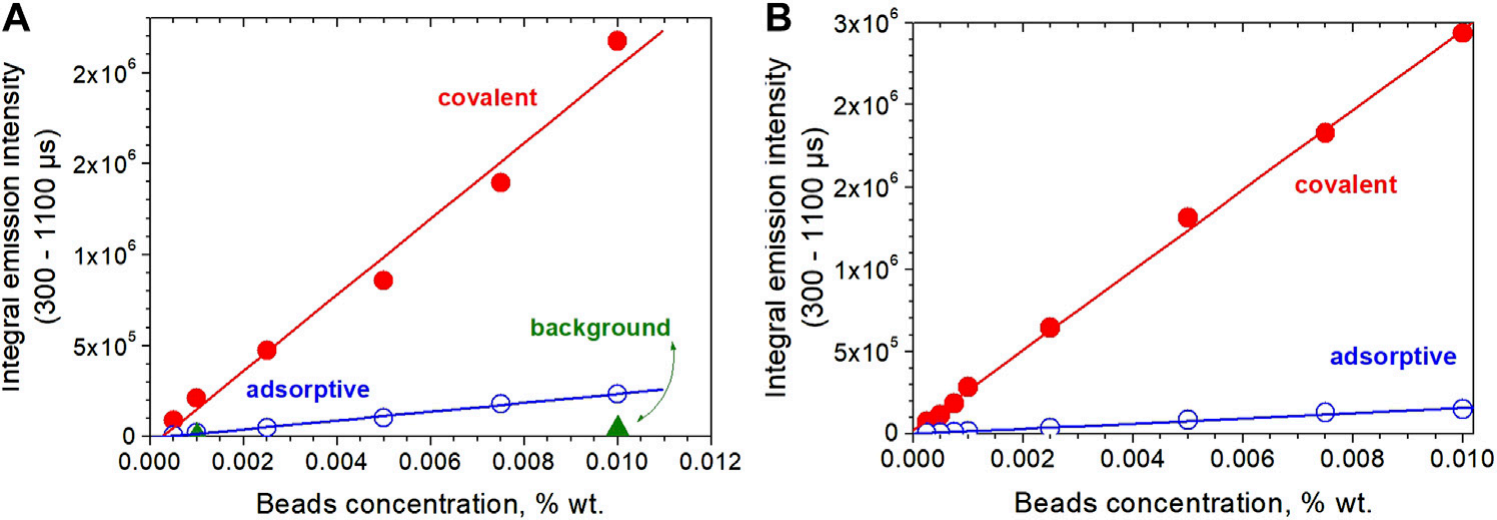
Time-gated luminescence intensity measurement of covalently and absorptively bead-coupled Neutravidin in biotinylated titerplates; volume added to vials was 200 µl. **(A)** Core-shell-beads from method 1 {[Eu(ttfa)_3_(TOPO)_2_/polystyrene/KPS]_core_[acrylic acid/methylmethacrylat/ACVA]_shell_)}; **(B)** core-shell-beads from method 2 {[Eu(ttfa)_3_(TOPO)_2_/polystyrene/styrene-4-sulfonate/KPS]_core_[acrylic acid//styrene-4-sulfonate/methylmethacrylat/KPS]_shell_}. Adsorptive means shelled beads without EDC/NHS-activation.

We tested two core-shell beads prepared by employing method 1 and method 2 (see previous paragraph). After covalent coupling and (unspecific) adsorptive coupling with Avidin or Neutravidin, to the shelled beads were tested in biotinylated titer plates by time-resolved determination in a Victor 4 spectrometer with time windows set between 300 and 1,100 µs. Figures 9A, B summarize the results for the core-shell-methods. The figures show the present limit to be 2.5 × 10^−4^% wt beads (ca. 1,000 beads/well), which conservatively recalculates into a 3 × 10^−18^ mol detection limit and even lower, if less than complete coverage of the bead surface with protein is required—which is most likely. Additionally, the amount of unspecifically adhering protein is between 11% (beads method 1) and 6% (beads method 2) only.

**FIGURE 10:**
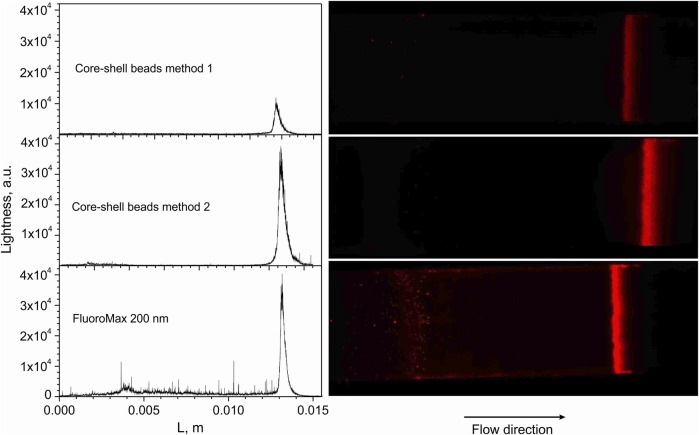
Lightness profiles and images from Lateral Flow strips along the chromatograpic flow direction from left to right. L is the approximate length from the chromatography start to the test line; the magnification was set to ×10, the exposure time was set to 10 ms. Further details on running media and microscopy may be found in the ESI.

**FIGURE 11:**
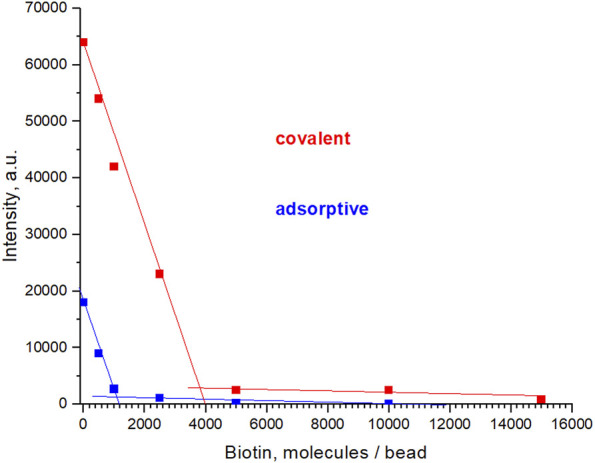
Fluorescence “Microscopy-Titration”: Results from lightness evaluation of the Biotin-testline of Lateral Flow strips after blocking the adherence of the analyte (Neutravidin) with increasing additions of Biotine prior to the chromatography. Both, Neutravidin covalently and adsorptively coupled are represented. Each data point corresponds to one individual chromatography experiment. Samples evaluated were core-shell particles after method 1. Microscope settings: excitation at 365 nm, emission 610 nm, 6% LED power, exposure time 50 ms. Further details on microscope and chemical procedures are given in the ESI.

Last but not least, an important test method in this context is the performance in Lateral Flow Immunoassays. Using the fluorescence microscope, we were able to follow the chromatography in LFIA strips. For this purpose, we used strips having a biotinylated test line to capture the Neutravidin-equipped beads; the more even and intense the response, the more useful the beads will be in LFIA. After “loading” of the strips with beads dispersed in a dedicated flow medium (running buffer), washing and drying, they were inspected microscopically and their relative lightness evaluated (the term lightness here meaning that the brightest sample signal was used to set the microscope settings below saturation of the camera, see ESI for details). Core-shell beads according to methods 1 and 2 and Fluoro-Max reference beads are reproduced in Figure 10. Beads from method 2 proved to be most superior: the lightness at the test line is almost identical to Fluoro-Max beads, but their trace is practically free from the “debris” seen in the Fluoro-Max chromatography, which obviously shows appreciable agglomeration in the running medium. It is obvious that method 1 had the weakest luminescence response in this experiment, but was almost free from left-behind debris. Hence, since exposure time and excitation intensity may be increased largely, the beads can still be useful. Finally, another independent and relatively simple semi-quantitative method—Fluorescence “Microscopy-Titration”—to estimate the Neutravidin-load of the beads was devised as follows: Avidin and Neutravidin are known to have 4 docking sites for Biotin. Hence, the addition of 4 equivalents molecular Biotin will completely block the protein sites such that the beads cannot adhere to the biotinylated test line on the Lateral Flow strip anymore. In other words, the intensity signal from the test line will decrease on incubating the test solution with higher concentration of Biotin, and the onset of signal permanence marks the Neutravidin saturation with Biotin as visible in Figure 11. The recalculation for beads from method 2 gave ca. 4000 Biotin molecules or roughly 1000 Neutravidin on the surface of the beads, i.e., the same order of magnitude as the HABA-titration above.

## 4 Conclusion

A plethora of rare earth complexes useful for immuno-assays have emerged during the last three decades, often with considerable preparative effort; originally employed, relatively simple β-diketonate complexes have long been outperformed by recent developments. Bottlenecks in the pursuit of suitable complexes were the screening against water and excitation energies compatible with suitable light sources, like high power UV-LEDs. While β-diketonates show a good match with, e.g., present 365 nm LEDs, their functionalization for subsequent conjugation to obtain biomarkers and protection from ambient water at the same time is not a trivial task. This had been realized at an early stage in this research already. Imbeddings into submicron-polymer particles soon proved to be an elegant solution to circumvent the drawbacks. Nowadays the toolbox for immunological beads contains various complexes and strategies for functionalization. We have ventured to directly compare very popular and efficient trifluoro-substituted, aromatic β-diketonates of Eu^3+^ with respect to their efficiencies as co-coordinated TOPO-complexes and in polymer beads; a novel aromatic system comprising 3-phenathrytrifluorodieketonate was also included. For methods with additive- and emulsifier-free polymer synthesis we found Eu(ttfa)_3_(TOPO)_2_ to be most suitable. We have therefore taken beads with occluded Eu(ttfa)_3_(TOPO)_2_ and elaborated two methods for applying shells in order to preserve the cores’ high efficiencies in aqueous dispersions and concurrently to provide anchoring groups for subsequent protein conjugation. The beads thus obtained were exemplarily tested for ELISA-like analyses and for Lateral Flow Immunoassyas; high-end commercial beads were compared as contol. The beads, taking advantage of a large amplification factors and ultimate brightnesses, enabled determinations in the attomol regime and beyond; in Lateral Flow experiments they proved to be superior to existing commercial materials accessible to us. With respect to efficiency, the beads are close to the physical limit. Further improvements in dispersions will presumably be restricted to the optimization of their scattering behaviour.

## Data Availability

The raw data supporting the conclusion of this article will be made available by the authors, without undue reservation.
