# Supplementary material for: High brightness red emitting polymer beads for immunoassays: Comparison between trifluoroacetylacetonates of Europium
Source: Front Chem. 2023 Apr 20;11:1179247. doi: 10.3389/fchem.2023.1179247 (PMC10157089; doi:10.3389/fchem.2023.1179247)
Supplement: Supplementary file 1 [file DataSheet1.pdf]

Supplementary Material

High brightness red emitting polymer beads for immunoassays: comparison between trifluoroacetylacetonates of Europium

Daniel K. Dinga, Ewa Kasprzycka, Israel P. Assunção, Franziska Winterstein, Amina Alizade, Volkan Caliskanyürek, Dirk Blödorn, Johannes Winkle, Ulrich Kynast* and Marina Lezhnina*

*** Correspondence:** Corresponding Authors: marina@fh-muenster.de, uk@fh-muenster.de

Content

1. **Apparatus**
   1.1. Spectroscopic mehods
   1.2. Quantum Yields
   1.3. Time-gated luminescence
   1.4. Fluorescence / Luminescence Microscopy
   1.5. Particle sizes
   1.6. ζ-potentials
2. **Materials and syntheses**
   2.1. Materials
   2.2. Syntheses and analyses
   2.3. Determination of complex contents within beads
   2.4. Coupling of core-shell carboxylate modified beads with Neutravidin (Avidin)
   2.5. Determination of surface carboxylate groups
   2.6. Determination of bound Neutravidin on beads
   2.7. Time gated analyses of beads on titer plates
3. **Chromatography on Lateral Flow strips with Biotin test-line**
   3.1. Microscopic flow monitoring on test strips
   3.2. Microscopy titration
4. **Supplementary Table. Analytikal data for the Eu(diketonate)$_3$(TOPO)$_2$-complexes**
5. **Supplementary Figures 1a–d (infrared spectra)**
6. **Supplementary Figure 2**
7. **References for Electronic Supplement**

1. Apparatus

1.1. Spectroscopic methods

Infrared spectra were recorded with a Spectrum One FTIR spectrometer (Perkin Elmer) as KBr pellets. Diffuse reflectance spectra used in the overall quantum yield determination of solid samples were recorded with an integrating sphere and the registered spectra corrected against $BaSO_{4(s)}$ as the white standard and active carbon as a black standard. The emitting state decay time measurements (τ) were performed in a Spectrofluorometer FS5, Edinburgh Instruments. The internal fitting procedures were used to obtain the decay times. Excitation

and emission spectra of the luminescent Eu^{3+} complexes were registered using Acton Research Corporation Spectra Pro®-300i monochromators equipped with a photomultiplier and a 450 W Xe-discharge lamp. The recorded excitation spectra were corrected against a Lumogen Red F300 doped (50 ppm) polymethyl methacrylate powder standard. UVVis-spectra were recorded with a SPECORD 200 Plus from Analytik Jena

1.2. Quantum yields

The quantum yields of all samples were measured at an excitation wavelength of 365 nm in powder form. Lumogen Red incorporated in PS polymer matrix was used as the reference (QY_{365nm}= 65%)[1]. For the oily $Eu(diketonate)_3(TOPO)_2$ complexes, a 1% wt solid mixture was prepared with KBr powder and properly homogenized. All measurements were done in a Teflon-lined integrating sphere using $BaSO_4$ and Carbon-black as white and black standards, respectively. The uncertainty of the reported data is estimated to be less than ±10 %.

1.3. Time gated luminescence measurements

Time gated measurements were performed on a Victor X4 multilabel plate reader (Perkin Elmer) in the time-resolved fluorometry mode using a UV-Xe flash- lamp, interference filter at 340 nm (bandwidth 10 nm) on the excitation side and an interference filter at 615 nm, bandwidth 8 nm) at the emission side.

1.4. Fluorescence / Luminescence Microscopy

Microscopy was done with a Leica DMi8 fluorescence microscope equipped with filtercubes, which allow specific imaging of the rare earth excitation/emission (Eu^{3+}: Excitation / FWHM = 365 nm/ 50 nm, dichroic mirror 420 nm, emission: 610 nm/ 30 nm) and a CoolLED pE-4000 LED light source and a Leica DFC9000TG digital camera. For brightness evaluation of image sections, the Leica standard software was used.

1.5. Particle sizes

Particle sizes were measured using a Nanotrac Wave II Q from Microtrac.

1.6. ζ-potentials

ζ-potentials were determined with a ZetaSizer Nano (Malvern) in a DTS1070 folded capillary cell.

2. Materials and syntheses

2.1. Materials

The chemicals and solvents were reagent grade and purchased from standard suppliers und used without further purification; as it turned out, styrene (99.5%) and methylmethacrylat (MMA, > 99%) from Carl Roth GmbH + Co. KG (Germany) did not have to be distilled for our syntheses. Toluene, petrol ether, tetrahydrofurane and dichloromethane, other solvents and MOPS buffer 3-(N-morpholino)propansulfonic acid) were purchased from Carl Roth GmbH + Co. KG and/or VWR-Avantor GmbH, both Germany. 4,4,4-trifluoro-1-(2-thienyl)-1,3-butandion (Httfa), 1-phenyl-4,4,4-trifluoro-1,3-butandion (Hbtfa), 4,4,4-trifluoro-1-(2-naphthyl)-1,3-butandion (Hntfa), Trioctylphospinoxide (TOPO, 99 %), aminoacrylate, p-aminostyrene and methacrylic acid, 2,2′-Azobis-(2-methyl-propionamidin)-dihydrochloride (AAPH), 2,2′-Azobis(2-Methylpropionitrile) (AIBN) and Potassium persulfate ($K_2S_2O_8$, KPS) were purchased from Merck (Sigma-Aldrich), 3-acetylphenanthrene (97%) from FisherScientific/Acros. HABA (4´-hydroxyazobenzene-2-carboxylic acid), Avidin, Neutravidin, Bovine Serum Albumin (BSA) and cationized BSA with an isoelectric point of > 10 (Imject™ cBSA (in PBS)) as well as biotinylated 8-well titerstripes were obtained from Thermo Fisher Scientific.

2.2. Syntheses and analyses

Synthesis of Hptfa (1-(3-phenanthryl)-4,4,4-trifluoroo-1,3-dione), Hptfa, $C_{18}H_{12}F_3O_2$

The synthesis of the ligand closely followed [2]: To 10 ml of anhydrous diethyl ether, 0.673 g (6.0 mmol) of anhydrous potassium tert-butoxide was added under $Ar_{(g)}$ flow while stirring. This was followed by the addition of 1.05 g (4.7 mmol) of 3-acetyl-phenanthrene. Then, finally, 0.71 g (5.0 mmol) of ethyl trifluoracetate dissolved in 5 mL of THF was added dropwise. The reaction mixture was allowed to stir overnight at room temperature. To the reaction mixture, 100 ml of 10% H_2SO_4(aq) was added and stirred for 10 minutes. This was followed by evaporation to remove diethyl ether. The resulting precipitate was then filtered and washed with distilled water and then recrystallized from CH_2Cl_2/petrol ether mixture and dried at 60 °C; yield 73 %. The structure was also confirmed with IR spectra (see Supplementary Figure 1a-d) below.

Preparation of the chelate complexes Eu(btfa)$_3$(TOPO)$_2$, Eu(ttfa)$_3$(TOPO)$_2$, Eu(ntfa)$_3$(TOPO)$_2$ and Eu(ptfa)$_3$(TOPO)$_2$.

The ‘binary’ Eu chelates Eu(btfa)$_3$(H_2O)$_2$, Eu(ttfa)$_3$(H_2O)$_2$, Eu(ntfa)$_3$(H_2O)$_2$ and Eu(ptfa)$_3$(H_2O)$_2$ were prepared essentially analogous to the procedure described in [3] using the ligand in ethanol and after deprotonation with aqueous NH_3 (1 molar) the addition of $EuCl_3(H_2O)_6$ in water. In the case of Eu(ptfa)$_3$(H_2O)$_2$, the $EuCl_3(H_2O)_6$ was co-solved with Hptfa in ethanol and deprotonated by the dropwise addition of exactly three eqivalents of aqueous NaOH (0.1 molar) under vigorous stirring.

The consecutive co-coordination with TOPO proceeded as described in [4] by dissolving the binaries in CH_3CN, followed by addition of 2 aliquots of TOPO and refluxing at 70 °C for 2 h to yield the ‘ternary’ complexes. For Eu(ptfa)$_3$(TOPO)$_2$, however, toluene was preferable, as in CH_3CN a notable TOPO ↔ CH_3CN ligand exchange was observed; the lower solubility of the binary in toluene was countered with prolonged reflux times. For cross-checking, Eu(ttfa)$_3$(TOPO)$_2$ was prepared via both routes prepared in this manner (CH_3CN or toluene): regardless of the solvent, the products were identical. Furthermore, in case of *Eu(ntfa)$_3$(TOPO)$_2$*, in the course of TOPO-coordination, a lightbrown phase separated from the CH_3CN solution, which was isolated and re-washed with a small amount of CH_3CN; the product obtained was then readily soluble in hexane. All complexes formed were thus isolated by evaporation of the solvents, re-dissolution in hexane, filtration of any precipitate (if present), evaporation of hexane and drying of the resulting oil in vacuum. Typical yields: binary complexes 65-75 %, ternary complexes > 80 %.
The binary complexes have been characterized analytically thoroughly in literature before, also by ourselves, hence we take the FTIR spectra, which are fully reproduced in Supplementary Figures 1a-d) as sufficient evidence for their identity; the optical spectra also comply with the literature.

Preparation of beads with Eu(diketonate)$_3$(TOPO)$_2$ (general procedure)

To compare the eventual performance of the beads, all ‘in situ’ syntheses (polymerization in the presence of complex) were conducted with 10% wt of complex with respect to styrene. Marginal deviations applied in the synthesis of Eu(ptfa)(TOPO)$_2$ are added in brackets. A two-neck flask with 70 ml of deionized water was warmed up to ca. 60 °C for mild degassing in a water bath and mixed with 35 ml methanol (ethanol). In a typical experiment, 170 mg of Eu(diketone)$_3$(TOPO)$_2$ complex in 1.6 ml of styrene were added to this mixture applying sonication, which was maintained for 5 min (20 min). The flask was equipped with a reflux condenser and placed into a pre-heated oil bath. Under magnetic stirring at 800 rpm, the temperature is maintained at 90 °C, and the set-up flushed with inert gas for 40 min. 0.200 g *2*,*2′*-azobis(*2*-methylpropionamidine) dihydrochloride (AAPH) was added to the emulsion,

after the polymerization start (clouding of the emulsion), the stirring rate was reduced 550 rpm and the polymerization continued for the next 5 h. The final bead dispersion was passeed through a Whatman 597 paper filter and separated by centrifugation at 21000 rpm, washed with water three times. Depending on the succeeding purpose, they were (vacuum-) dried at 60 °C overnight, e.g. to determine the solid content of the dispersion gravimetrically or spectroscopies. Complete redispersion of the centrifuged beads was accomplished for non-dried samples only; dispersions for subsequent experiments could be stored at 4 °C over months, but had then to be agitated roughly (e.g. ultrasonically) to re-disperse sediments. The content of solids in the dispersions was determined gravimetrically.

Preparation of Eu(ttfa)$_3$(TOPO)$_2$-beads with surface-amino-groups

The procedure proceeded in analogy to the previous synthesis, with the exception that after stirring of the AAPH-initiated reaction for 1 hr at 90 °C, 50 mg of aqueous 2-aminoethylmethylacrylate hydrochloride (AEMH) were added. The work-up was the same as described above. Particle sizes amounted to an average of 166 nm, the ζ-potential was measured to be +39 mV. The decays were fitted as biexponential on average with τ_1 = 607 µs (73 %) and τ_2 = 977 µs (27 %). The variation between different batches amounted to less than 3 %. The dispersion exhibited 86 % of the brightness of a commercial product (Fluoro-Max / Fisher Scientific, 200 nm, carboxylated, ca. $5x10^5$ -COOH/particle).

Preparation of monodisperse core-shell beads with Eu(ttfa)$_3$(TOPO)$_2$: (Method 1)

The following description yields beads with carboxylated surfaces:
170 mg of Eu(ttfa)$_3$(TOPO)$_2$ were dissolved in 1.6 ml styrene. A 250 ml two-necked flask, with 60 ml of deionized water was heated to 60 °C in a (quiet) ultrasonic bath, to which 40 ml of methanol were added; ultrasonication was started and the styrene solution with the complex added within a few seconds. After 5 minutes, the mixture became turbid. The flask was transferred to an oil bath, pre-heated to 90 °C, equipped with a reflux condenser, flushed with inert gas and stirred vigorously with a magnetic stirrer (800 rpm) for 40 min, on which the turbidity faded (see previous protocol). To initiate polymerization, 100 mg powderous KPS ($K_2S_2O_8$) are added using a funnel. After the mixture turns cloudy, polymerization is continued for 1 hr at a reduced stirring rate of 550 rpm. The shell components, 0.208 ml acrylic acid, 0.232 ml methylmethacrylate in 1 ml methanol and 3.03 ml 1 M NaOH, diluted with 6 ml 50 mM HEPES buffer (pH 7.3), are poured into a dropping funnel with pressure compensation. The funnel is placed on the second neck of the reaction vessel under inert gas flow. After 1.5 hrs – concluded core-formation – the shell components are added dropwise (0.5 ml/min). 60 mg ACVA-initiator (4,4′-azobis-(4-cyanovaleric acid)) are then added, again via a funnel, and neutralized instantly with 0.214 ml 1 M NaOH. The polymerization is continued for another 4 hrs. The bead dispersion was filtered through a medium pore size paper filter Whatman 597, after which the beads were isolated by centrifugation at 21000 rpm, washed with water and dried at 60 °C overnight. Only part of product was dried, the residue was re-dispersed in water and stored at 4 °C for follow-up experiments. The contents of solids in the resulting dispersions were determined gravimetrically. The total of 1.068 g beads corresponded to a yield of roughly 50 %. An electron microscopy image of the beads is presented in Figure 8 of the main text. The amount of shell starting materials there corresponds to 27.5% wt with respect to styrene.
A modification of method 1 to yield an increase of the complex content to 13.3 % could be accomplished using 20% wt of Eu(ttfa)$_3$(TOPO)$_2$ initially, by increasing the amount of methanol, KPS initiator for core and shell, as well as a longer core-formation time (4 hrs). Thinner shells could be prepared in the same manner using 10% wt of the shell material (5% acrylic acid, 5 % methylmethacrylate). However, this procedure went at the cost of agglomerated particles and a bimodal distribution (number distribution 334 nm (69 %) and 147 nm (31%)), see Supplementary Figure 2 for spectra. Optical data: a) Core only - quantum

yield 79 %, 1-Ref_{365} (absorption) > 0.95, decay time τ = 645 µs; b) core-shell - quantum yield 95 %, 1-Ref (absorption) > 0.95, decay time τ = 652 µs. The ζ-potential for the core-shell-particles was -38.6 mV.

Preparation of monodisperse core-shell beads with $Eu(ttfa)_3(TOPO)_2$: (Method 2)

25 mg styrene-4-sulfonic acid sodium salt (NaSS), 2% wt relative to styrene, was dissolved in 27 ml 50 mM MOPS buffer (pH 7.2), preheated in a three neck flask to 70 °C and mixed with 37 ml methanol. 1.36 ml complex solution (22.6% wt) in styrene (280 mg in 1.36 ml styrene) was added to this mixture under sonication.

The mixture was sonicated for the next 5 min under sonication, then placed into a pre-heated oil bath (90 °C) and stirred (550 rpm) for 40 min under reflux and inert gas atmosphere. 0.200 g KPS were dissolved in 5 ml of water and added to the emulsion and polymerization continued for 1.5 h. A mixture of 0.059 ml acrylic acid (5% wt relative to styrene) diluted with 2 ml MOPS buffer, 0.859 ml 1M NaOH and 25 mg NaSS (2% wt relative to styrene) dissolved in 2 ml MOPS buffer, 1 ml methanol and 0.066 ml methylmethacrylate (5% wt relative to styrene) were slowly dropped to the reaction mixture (0.3 ml/min). 60 mg KPS dissolved in 2 ml dest water were added immediately before and after addition of the shell monomers and the polymerization continued for the next 4.5 h. The bead dispersion was filtered through medium pore paper filter Whatman 597, the beads were isolated by centrifugation at 21000 rpm, washed with water. Only part of the product was dried at 60 °C overnight; the remainder was re-dispersed in water and stored at 4 °C for follow-up experiments. The content of solids in the dispersions was determined gravimetrically.

2.3. Determination of complex contents within beads

The amount of luminescent complex incorporated into the polymer matrix (denoted as the Eu-content of the beads) was investigated by standard addition analysis of the Eu emission signal. A test solution PS-Eu beads in THF was prepared with a loading of 140 mg/L. This solution was diluted to an absorbance value ≤ 0.15 (solution x whose concentration of Eu^{3+} $[S]_x$ will be determined). The Eu^{3+} emission intensity was measured and noted. Then a standard solution of corresponding complex in THF was prepared with a concentration of 0.05 – 0.1 mM. Subsequently, a concentration series was prepared by adding 200 µL of the standard solution to 2.5 mL of the test solution. The Eu^{3+} emission intensity was measured after each addition. The concentrations of added Eu-complex in these solutions [S] were plotted against the emission intensities of the solutions and the unknown concentration $[S]_x$ was extrapolated from the data and the actual Eu^{3+}-content calculated.

2.4. Coupling of core-shell carboxylate modified beads with NeutrAvidin (Avidin)

Activation of carboxylic groups

1 ml 1% wt of Eu-PS-COOH bead dispersion (for example $Eu(ttfa)_3(TOPO)_2$, 20% wt of complex , method 1) were centrifuged, washed three times with 50 mM MES buffer of pH 5.8 and re-dispersed in an equal amount of MES buffer.

120 µl of 0.1 M *N*-(3-dimethylaminopropyl)-*N′*-ethylcarbodiimide hydrochloride (EDC) solution in dry DMSO and 140 µl 0.5M N-hydroxysuccinimide (NHS) solution in dry DMSO were added to 1 ml of a 1% wt bead-dispersion in MES-buffer; the mixture was shaken for 15 min at room temperature and centrifuged. The activated beads were washed with 1 ml MES buffer of pH 5.8 and re-dispersed in 500 µl of 50 mM MOPS-buffer of pH 7.2 and used for coupling with proteins immediately.

Coupling

300 µl of activated bead dispersion were mixed with 800 µl Neutravidin or Avidin solution with a concentration of 0.2 mg/ml. The reaction mixture was shaken for 2.5 h at room temperature. Unreacted active groups were deactivated by addition of 80 µl 1M Gly-Gly

(diglycine) buffer at a pH of 7.5. The protein-coupled beads were isolated via centrifugation, washed with BSA-MOPS buffer (50mM MOPS pH 7.2, 1% wt BSA, 0.02% wt sodium azide) three times, re-dispersed in 600 µl BSA-MOPS buffer; storage at 4 °C.
It proved to be advantageous, to subject the bead-protein assembly to further washing steps (up to three times) with 0.1% wt solution of positively charged BSA (cationic cBSA, i.e.p. > 10) in 50 mM MOPS buffer at a pH of 7.2, washed again with BSA-MOPS buffer, isolated and re-dispersed in BSA-MOPS buffer. For comparison, samples with Neutravidin (Avidin) only adsorbed rather than covalently conjugated to the bead surface were prepared. For this purpose the beads with the same treatment but without activation were used.

2.5. Determination of surface carboxylate groups

2 ml of 1% wt activated beads (method 1; EDC/NHS, as described above, 'Activation of beads') in MOPS were mixed with 36 µmol of hexylamine (4.73 µl) and reacted for 3 h at r.t. The beads were centrifuged off. Mother liquor and washing liquid were then titrated with o-phthalaldehyde as described in the literature [5]. The recalculation yielded 458000 COOH/bead and a parking area of 25 $Å^2$.

2.6. Determination of bead-bound Neutravidin

The analytical procedure for Avidin analysis using 4´-hydroxyazobenzene-2-carboxylic acid (HABA) applied is described in [6, 7]. A calibration curve was established by stepwise addition of 400 µl NeutrAvidin solution with known concentration (0.0168 – 0.168 mg/ml) to 1.6 ml of a 0.25 mM solution of HABA and estimation of the absorption difference between this solution and the same solution in the presence of 5 mM Biotin at 500 nm (9 data points, R^2 = 0.9979). Core-shell beads from method 1 thus bore 2280 molecules/bead (59.3 mg/g beads), which is fairly close to the maximum amount of 2674 molecules/ bead, assuming a hydrodynamic diameter of 7.4 nm for Neutravidin [8].

2.7. Time gated analyses of beads on titer plates

The wells of a titer plate (8 well strip) were rinsed three times with 300 µl of BSA-MOPS-buffer (see above, 'Coupling'). Subsequently 200 µl of the bead-Neutravidin sample solutions were incubated for 1 h. The solution was then removed and the wells again washed three times with 300 µl of the BSA-MOPS-buffer. Time gated luminescence was determined within a time window of 300-1100 µs.

3. Chromatography on Lateral Flow strips with Biotin test-line

3.1. Microscopic flow monitoring on test strips

Cellulose nitrate membrane strips CN-140 with a biotinylated test line and absorption pad (generously provided by R-Biopharm, Darmstadt, Germany) were used for the comparison of adsorbed and covalently conjugated coupled Neutravidin beads.
50 µl 0.0025% wt bead dispersion in running buffer with 50 mM Gly-Gly (diglycine) pH 7.5, 0.9% wt NaCl, 1% wt BSA, 1% wt Brij35) were placed into a 96-well titer plate. The test strip was put into the dispersion for 5 min, removed and the absorption pad put into the well with 50 µl pure running buffer for 5 min. The strips were dried in a vertical position and analyzed under the fluorescence microscope. Acquisition parameters: objective lens 10x, excitation filter 350/50 nm, dichroic mirror 420 nm, emission filter 610/30 nm, exposure time fluorescence – 10 ms, scanning and stitching of 70 tiles from start position to the test line.

3.2. 'Microscopy titration'

For each of the 'Microscopy titration' steps the same procedure as described above was employed, now on membrane strips CN-95 with biotinylated test line (R-Biopharm). The desired amounts of Biotin were added prior to the flow.

4. Supplementary table: Analytikal data for the Eu(diketonate)$_3$(TOPO)$_2$-complexes

Complex		Eu(ttfa)$_3$(TOPO)$_2$	Eu(btfa)$_3$(TOPO)$_2$	Eu(ntfa)$_3$(TOPO)$_2$	Eu(ptfa)$_3$(TOPO)$_2$
Carbon	theoretical	54.43	59.53	62.7	65.47
	experimental	55.00	58.76	68.35	66.60
Hydrogen	theoretical	7.23	7.87	7.54	7.13
	experimental	-	-	6.77	8.30
Eu	theoretical	9.56	9.66	8.81	8.12
	experimental	9.80	9.93	7.62	7.05

5. Supplementary Figures 1a–d (infrared spectra)

Supplementary Figure 1a). IR-spectroscopy for the ligands, the binary and TOPO-co-coordinated Eu(ptfa)$_3$(TOPO)$_2$ (ptfa = 3-phenanthryl-4,4,4-trifluoro-1,3-dionate (see main text Figure 1 for structure).

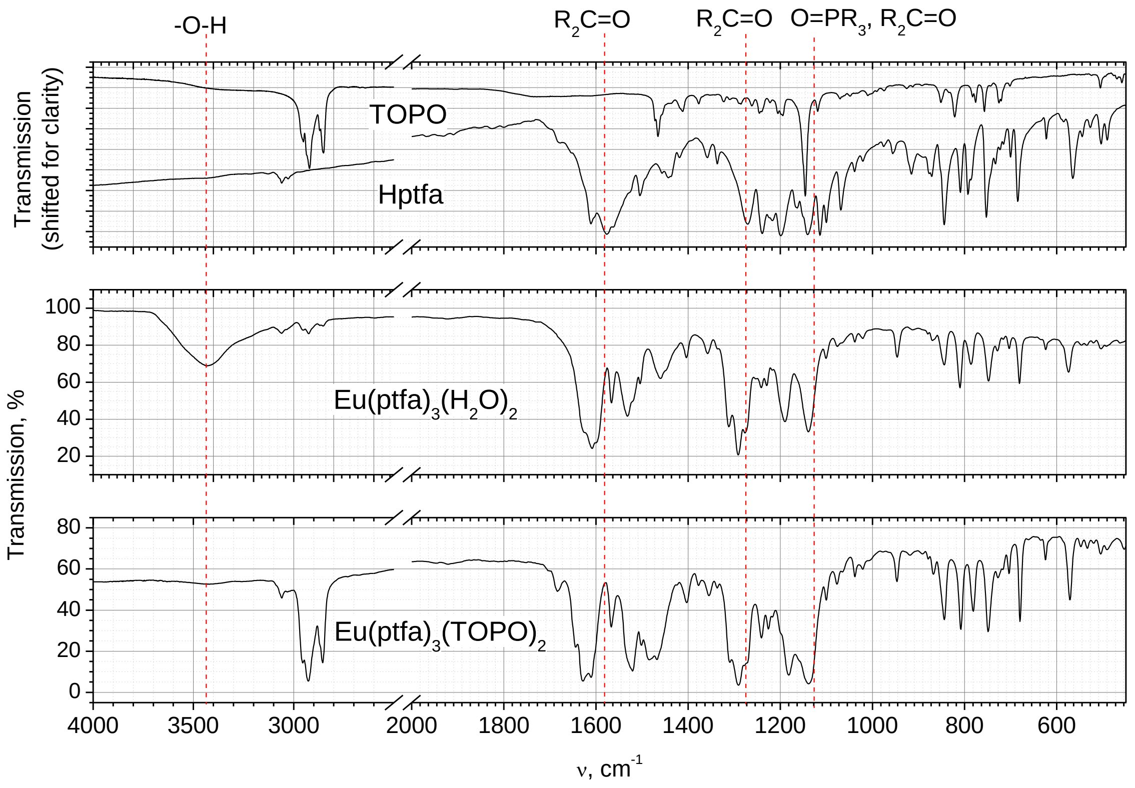

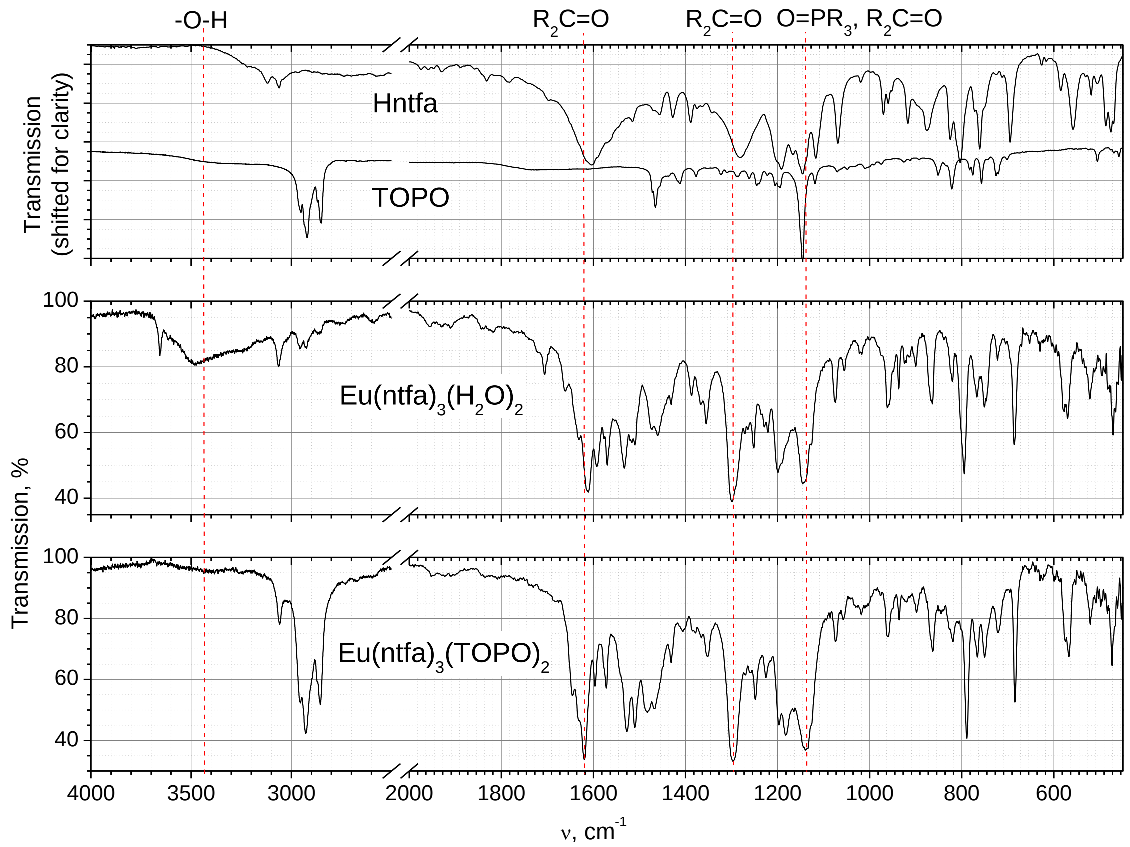

Supplementary Figure 1b). IR-spectroscopy for the ligands, the binary and TOPO-co-coordinated Eu(ntfa)$_3$(TOPO)$_2$ (ntfa = 2-naphthyl-4,4,4-trifluoro-1,3-dionate (see main text Figure 1 for structure).

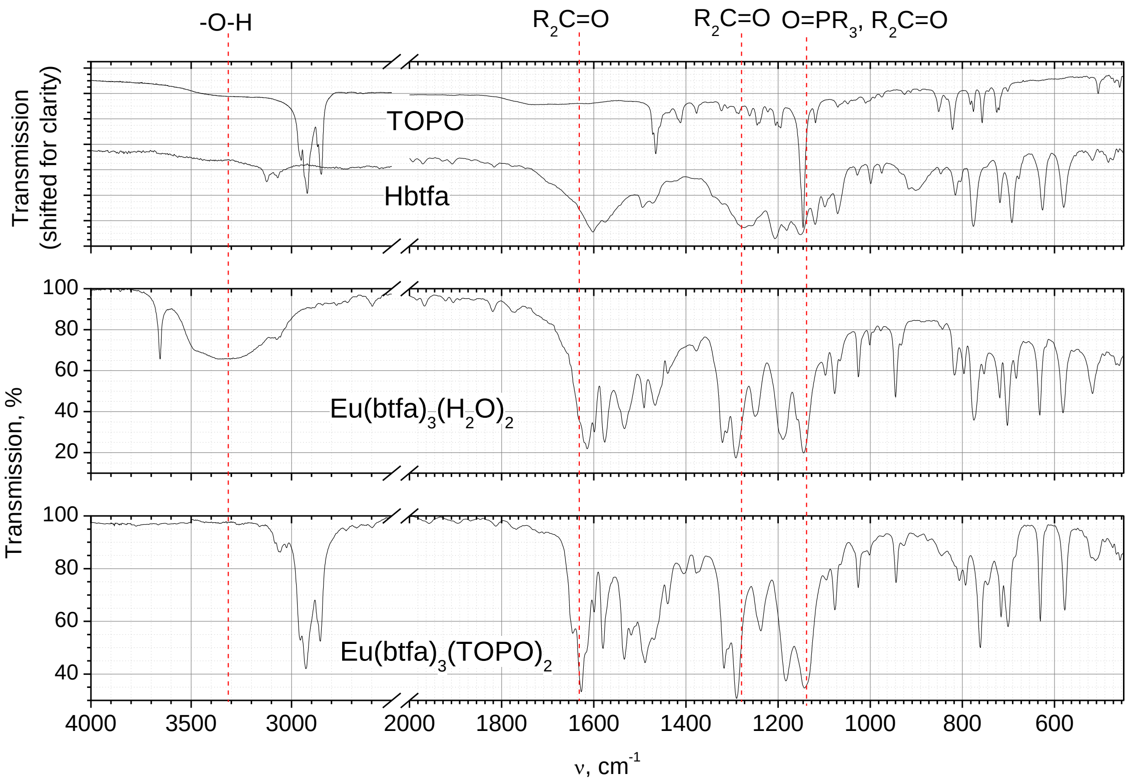

Supplementary Figure 1c). IR-spectroscopy for the ligands, the binary and TOPO-co-coordinated Eu(btfa)$_3$(TOPO)$_2$ (btfa = 1-phenyl-4,4,4-trifluoro-1,3-dionate (see main text Figure 1 for structure).

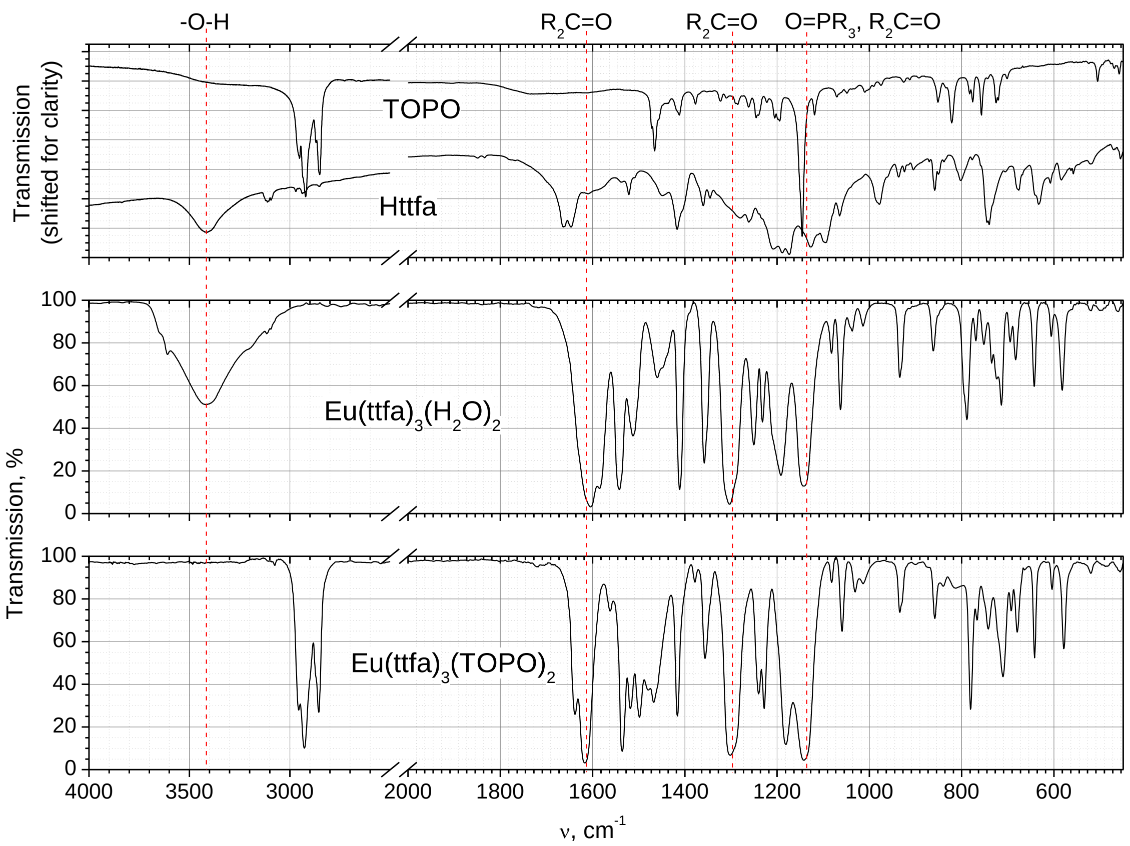

Supplementary Figure 1d). IR-spectroscopy for the ligands, the binary and TOPO-co-coordinated Eu(ttfa)$_3$(TOPO)$_2$ (ttfa =2-thienyl-4,4,4-trifluoro-1,3-dionate (see main text Figure 1 for structure).

6. Supplementary Figure 2

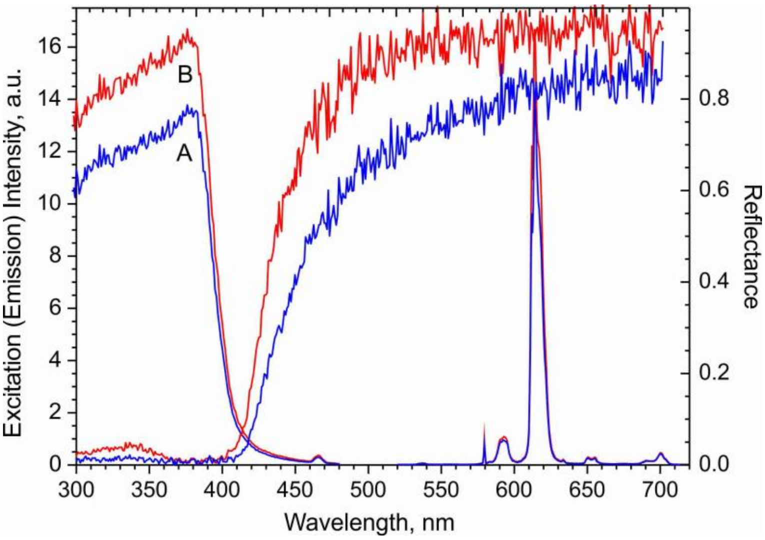

Supplementary Figure 2. Luminescence and reflectance spectra for pure core (A) and modified shell synthesis (method 1) (B). Synthesis of both with 20% wt Eu(ttfa)$_3$(TOPO)$_2$; 13.3% wt incorporated, see above 'Preparation of monodisperse core-shell beads with Eu(ttfa)3(TOPO)2' and 'A modification of method 1' above also.

7. References for Electronic Supplement

1. Lezhnina, M.M., Kaetker, H., Kaiser, M., Stegemann, L., Voss, E., Resch-Genger, U., et al. (2016). Chemical behavior and spectroscopic properties of rare earth borates in glazes. J. Lumin. 170, Part 2, 387-394.
2. Dinga, D., Lezhnina, M., Alizade, A., and Kynast, U. (2023). High Efficiency Luminescent Polystrene Beads with a Novel Europium Phenanthrene Diketonate. Opt. Mater. xxx, xxx-xxx, https://dx.doi.org/10.2139/ssrn.4364461.
3. Charles, R.G., and Ohlmann, R.C. (1965). Europium thenoyltrifluoroacetonate, preparation and fluorescence properties. J. Inorg. Nucl. Chem. 27(1), 255-259.
4. Tran, T.H., Bentlage, M., Lezhnina, M.M., and Kynast, U. (2014). Efficient Red Emission from Europium Chelate-Silicone Host-Guest Hybrids. Z. Naturforsch. B 69b(2), 210 – 216.
5. Roth, M. (1971). Fluorescence reaction for amino acids. Anal. Chem. 43(7), 880-882.
6. Fisher Scientific. (2023). Instructions HABA (4'-hydroxyazobenzene-2-carboxylic acid) (accessed Feb., 28th, 2023). Available from: https://www.thermofisher.com/order/catalog/product/de/de/28010.
7. Green, N.M. (1970). Spectrophotometric determination of avidin and biotin. Methods. Enzymol. 18 (a), 418-424.
8. Langer, A., Kaiser, W., Svejda, M., Schwerter, P., and Rant,U. (2014). Molecular Dynamics of DNA–Protein Conjugates on Electrified Surfaces: Solutions to the Drift-Diffusion Equation. J. Phys. Chem. B 118(2), 597-607.
